# Exploring the impact of psychological capital and work–family conflict on stress regulation and success in competitive athletes

**DOI:** 10.3389/fpsyg.2025.1566508

**Published:** 2025-10-20

**Authors:** Bing Bai, Rui Ma, Jabbar Rehima

**Affiliations:** ^1^Physical Education Department, Jiangxi University of Water Resources and Electric Power, Nanchang, Jiangxi, China; ^2^Physical Education School of Zhengzhou University, Zhengzhou, Henan, China; ^3^Department of Sport Education, College of Education, The Islamic University, Najaf, Iraq; ^4^Department of English Language, College of Education, The Islamic University of Al Diwaniyah, Al Diwaniyah, Iraq; ^5^Department of English Language, College of Education, The Islamic University of Babylon, Babylon, Iraq

**Keywords:** psychological capital, work–family conflict, perceived stress, athletic success, sports success

## Abstract

The psychological well-being of athletes is influenced by multiple interconnected factors that can significantly impact their performance outcomes. This study examined the predictive relationships between psychological capital, work–family conflict, and perceived stress in determining athletic success. We investigated how these psychological constructs influence performance outcomes among professional athletes. The combined sample comprised 240 professional athletes who completed validated measures of psychological capital, work–family conflict, and perceived stress. Discriminant analysis was employed to identify which variables most effectively differentiated between successful and less successful athletes. Results revealed that psychological capital dimensions and work–family conflict significantly predicted athletic performance outcomes. Stepwise discriminant analysis identified optimism, self-efficacy, hope, work–family conflict, and resilience as the strongest predictors of athletic success. The findings demonstrate that work–family conflict serves as a critical mediating factor between psychological capital and athletic performance, with increased conflict associated with elevated stress levels and diminished psychological resources. These results highlight the importance of considering athletes’ broader life context when examining performance predictors. The study contributes to understanding how personal psychological resources interact with external stressors in the athletic domain, suggesting that interventions addressing both individual psychological capital and work-family balance may be most effective in supporting athletic achievement.

## Highlights

- Psychological characteristics influence success in sports and stress management.
- Work–family conflict impacts athletes’ stress and psychological capital significantly.
- Optimism, self-efficacy, and resilience strongly predict sports performance outcomes.
- Family dynamics and stress are key factors in athletes’ success or failure.
- Stress management and family awareness are vital for supporting athletes’ success.

## 1 Introduction

One crucial element in sports that drives motivation, particularly among youth, is “championship sports.” Achieving titles in prestigious national and international events, which capture the attention of many nations, can significantly impact a society’s economic, cultural, and social status. Consequently, many countries invest in meticulous planning to train and prepare athletes for success in major competitions (Jeiharan, 2023). The multifaceted nature of athletic success extends beyond mere physical preparation, encompassing complex psychological processes and social dynamics that collectively determine performance outcomes in competitive environments (Jeiharan, 2023; Sanıoglu et al., 2017). Championship sports also serve as a powerful symbol of national identity and unity. Victories in international tournaments often evoke strong feelings of pride and solidarity, reinforcing cultural cohesion and enhancing a nation’s visibility on the global stage (Mutz and Gerke, 2024). Moreover, success in these arenas can create role models who inspire younger generations to pursue athletic careers, fostering a continuous cycle of talent development. From an economic perspective, sporting achievements can attract sponsorships, investments, and tourism, thereby stimulating local and national economies. In this sense, championship sports function not only as a vehicle for personal glory but also as a catalyst for broader socio-economic growth. Equally important is the recognition that elite performance is shaped by a synergy of multiple factors. While physical training and technical skill are essential, psychological resilience, motivation, and the ability to cope with stress are decisive in high-pressure competitions. Social support networks, including coaches, peers, families, and broader institutional structures, also play a pivotal role in sustaining athletes throughout their careers. The integration of sports science, nutrition, biomechanics, and advanced data analytics further illustrates the interdisciplinary nature of success in championship sports. Thus, preparing athletes for such high-stakes contexts demands a holistic strategy that balances physical, mental, and social dimensions to maximize both performance and long-term well-being.

### 1.1 Theoretical framework

Contemporary sports psychology literature suggests that athletic success is best understood through an integrated theoretical framework that considers the interplay between individual psychological resources, environmental stressors, and social role management. This study builds upon three foundational theoretical perspectives: Conservation of Resources Theory (Hobfoll, 1989), Work-Family Border Theory (Clark, 2000), and Positive Psychology Theory (Seligman, 2011), to develop a comprehensive understanding of championship athletic performance.

Conservation of Resources Theory provides the underlying mechanism explaining how athletes manage their psychological resources under competitive stress. According to this theory, individuals strive to obtain, retain, and protect resources, and stress occurs when these resources are threatened or depleted (Hobfoll, 1989). In the athletic context, psychological capital represents a crucial resource pool that athletes can draw upon to maintain performance under pressure. Work-Family Border Theory offers insights into how athletes navigate the boundaries between their professional sporting commitments and personal life demands, suggesting that successful boundary management is essential for optimal performance (Clark, 2000). Finally, Positive Psychology Theory emphasizes the role of positive psychological states such as hope, optimism, resilience, and self-efficacy in enhancing human performance and well-being (Seligman, 2011).

### 1.2 Psychological stress and athletic performance

Athletic performance and success are heavily influenced by various stress factors inherent in sports. Psychological stress can arise from mental errors, physical changes, health issues, high expectations, intense competition, professional and personal threats, pain tolerance, witnessing competitors’ successes or cheating, and the imbalance between personal and professional life (Jeiharan, 2023; Sanıoglu et al., 2017). The literature distinguishes between different types of stress responses, with eustress (positive stress) potentially enhancing performance and distress (negative stress) typically impairing it. This nuanced view aligns with research in organizational settings, which demonstrates that the experience of job stress is not universally negative and can, under certain conditions, act as a catalyst for producing innovative ideas and solutions (Li J, et al., 2025).

However, existing research reveals significant gaps in understanding how athletes’ stress responses vary across different demographic characteristics, cultural contexts, and competitive levels. While general stress measurement tools like the Perceived Stress Scale (PSS) provide valuable insights, they may not capture the sport-specific stressors that uniquely affect athletic performance. Furthermore, the interaction between perceived stress and other psychological variables remains underexplored, particularly in championship-level competition where stress levels are typically elevated. The capacity to navigate this high-stress environment may be linked to cross-cultural adaptability and emotional regulation, competencies which are not always directly correlated; for instance, higher emotional intelligence does not automatically guarantee superior cultural intelligence in managing novel or intercultural stressors (Li Y, et al., 2025).

Identifying and evaluating key psychological indicators in young athletes, such as anxiety levels, coping skills, and mental capabilities, is valuable for coaches, families, and parents. This awareness helps prevent the misallocation of an athlete’s energy and talent and guides their development along the most suitable path (Chen and Mok, 2024). Research has demonstrated that emotional traits, perceived stress, and anxiety are linked to increased blood pressure (Farahani et al., 2019), which can detract from performance, particularly at professional and championship levels (Miçoogullari and Ekmekçi, 2017).

### 1.3 Psychological capital in athletic contexts

Studies on championship and Olympic athletes indicate that mental training is an essential component of their regimen, helping to alleviate stress and mental pressure (Schinke et al., 2018; Talha, 2023). Gould et al. (2001) found that athletes who developed mental skills during training performed better in terms of behavior and sports outcomes compared to their peers. Positive behavioral traits, such as effective tension management, control over negative emotions, high motivation, and strong self-confidence, are crucial for success in high-stakes competitions. These skills not only enhance physical performance but also contribute to mental stability, preparing athletes to handle intense competitive pressures (Gould et al., 2001). This mental stability is underpinned by a strong ethical and moral foundation, which recent research suggests is integral to developing functional attitudes that support long-term well-being and adaptive social functioning, even in high-pressure contexts (Lyu et al., 2025).

Psychological capital, conceptualized as a higher-order construct comprising hope, optimism, resilience, and self-efficacy, represents a crucial psychological resource that enables athletes to maintain stability and motivation amidst challenges and stress from competitions effectiveness (Luthans and Youssef-Morgan, 2017). This concept refers to an individual’s self-perception of their inner abilities and capacities, reflecting their belief in their own skills and competencies (Hosaini Nia et al., 2015; Lee et al., 2017; Yildiz, 2018). This adaptive capacity and belief in one’s ability to overcome challenges may be rooted in neurobiological processes, with insights from neuroscience and molecular genetics supporting the adage that exposure to manageable stressors can ultimately foster greater strength and resilience (Gan et al., 2024). Athletes with high psychological capital not only trust in their technical and physical abilities but also in their capacity to effectively utilize additional resources like financial, human, and social capital (Lee et al., 2017; Lee et al., 2022).

However, the literature reveals limited understanding of how psychological capital functions across different demographic groups, competitive levels, and cultural contexts. The absence of analysis regarding gender differences, age variations, and educational background represents a significant gap in current knowledge. These demographic factors may moderate the relationship between psychological capital and athletic performance, suggesting that a one-size-fits-all approach to psychological preparation may be insufficient. Such demographic moderators extend beyond age and gender to include factors like educational attainment, which has been shown to significantly influence fundamental personal and familial attitudes, such as parental gender preferences (Fei and Li, 2025).

### 1.4 Works-family conflict in athletic careers

Fletcher and Scott (2010) highlight that occupational and organizational stressors are significant sources of stress for educators, with these factors interacting to exacerbate mental pressure. This increased stress can negatively impact the performance and success of coaches and their teams (Fletcher and Scott, 2010). Zheng and Wu (2018) describes work–family conflict as a misalignment between work demands and family life expectations. Such conflicts make it challenging to manage both areas simultaneously, leading to stress and decreased quality in professional and personal life (Zheng and Wu, 2018). This challenge is compounded by the finite nature of time, where intensive athletic schedules inevitably infringe upon time otherwise allocated to family roles, a dynamic shown to be a key determinant of overall family well-being (Hong et al., 2024).

The unique demands of athletic careers including extensive travel, irregular schedules, intense training regimens, and competitive pressures create distinctive challenges for work-family balance. Unlike traditional occupations, athletic careers often require sacrifices in personal relationships, family time, and social activities, potentially creating conflicts that extend beyond typical work-family boundaries. Hosaini Nia et al. (2015) reports that family and coach support can mitigate work–family conflict and reduce burnout among employees and athletes. Enhanced support from supervisors and family can lead to better performance and the emergence of talents in competitive environments (Hosaini Nia et al., 2015). Clark (2000) conducted a foundational study examining the impact of workplace transformations on work-life balance. Their findings demonstrated that socio-cultural changes and increasing work demands lead to imbalance in employees’ professional and personal lives. The researchers discovered that growing social awareness, particularly among women, and changing family structures have created additional challenges in managing work and family responsibilities simultaneously (Clark, 2000). Kim et al. (2017) conducted a study examining the role of psychological capital (PsyCap) among sports employees on attitudes (job satisfaction and psychological well-being), behaviors (organizational citizenship), and performance. The findings demonstrate that developing PsyCap can serve as a key strategy for promoting positive organizational behavior in sports environments (Kim et al., 2017). Subsequent studies, such as the research by Smith (2020), confirmed that escalating stress levels and workplace pressures significantly increase employee turnover rates. These researchers concluded that progressive organizations need to create more flexible work environments to mitigate these negative effects. The findings of these studies provided a solid scientific foundation for developing human resource policies aimed at improving work-life balance (Smith, 2020). Sarwar et al. (2021) conducted a study examining the impact of occupational and psychological factors on work-family balance satisfaction among academic faculty members. The findings revealed that job demands decrease balance satisfaction by increasing conflict and reducing work-to-family enrichment, whereas job resources enhance satisfaction by strengthening psychological capital and improving enrichment. This research emphasizes the importance of simultaneously considering both organizational and individual factors in improving the quality of work life for university professors (Sarwar et al., 2021).

**Figure 1:**
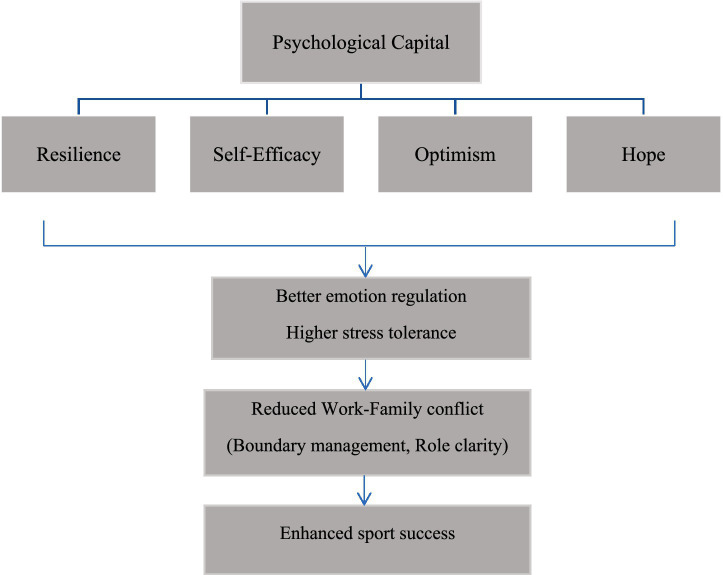
Psychological capital and work–family conflict conceptual model.

Despite its apparent importance, work–family conflict remains an understudied variable in sports psychology research. The mechanisms, through which work–family conflict influences athletic performance, and its interaction with psychological capital, require further investigation. This gap is particularly significant given the growing recognition that athletic success depends not only on individual psychological resources but also on the athlete’s ability to manage multiple life domains effectively. Figure 1 shows a model of the role of psychological capital and work–family conflict in athletic success.

As illustrated, psychological capital comprising resilience, self-efficacy, optimism, and hope functions as an internal resource that enhances emotion regulation and stress tolerance. These improved psychological capacities reduce work–family conflict through more effective boundary management and role clarity. Consequently, athletes benefit from greater mental focus and emotional stability, which ultimately lead to enhanced sport success.

### 1.5 Integration and research gaps

The literature reveals several critical gaps that limit our understanding of championship athletic performance. First, the majority of studies examine psychological variables in isolation, failing to consider their interactive effects and the complex pathways through which they influence performance. Second, the lack of sport-specific measurement tools may result in incomplete assessment of relevant psychological constructs. Third, demographic and cultural factors are often overlooked, despite their potential moderating effects on psychological processes. Fourth, the role of work–family conflict as a mediating or moderating variable in the relationship between psychological capital and athletic performance remains largely unexplored.

Bhat (2017) found a positive correlation between psychological capital and sports success in coaches (Bhat, 2017). Luthans and Youssef-Morgan (2017) demonstrated that enhancing positive psychological traits, particularly psychological capital, significantly boosts job satisfaction and work performance. Psychological capital improves positive attitudes toward oneself and the work environment, leading to increased productivity and effectiveness (Luthans and Youssef-Morgan, 2017). Dirmanchi and Khanjani (2019) highlighted a significant relationship between resilience and self-efficacy with exercise, suggesting that exercise can enhance these traits in individuals with spinal cord amputations (Dirmanchi and Khanjani, 2019).

### 1.6 Study objectives

Research on championship sports psychology is limited, and investing in this field is essential for developing national and international sports heroes. Identifying psychological factors that influence sports behavior and success is crucial for effective sports management. Examining variables affecting athlete performance is vital for achieving sports success. This research addresses the identified gaps by developing and testing an integrated model that examines the relationships between psychological capital, work–family conflict, perceived stress, and athletic performance. While acknowledging the limitations inherent in secondary data analysis including potential methodological differences between source studies, varying cultural contexts and limited control over data collection conditions this study provides valuable insights into the complex psychological processes underlying championship athletic success.

The distinction of this study lies in its exploration of work–family conflict as a crucial component in the relationship between psychological capital and sports success or heroism. Unlike other studies, this research provides a comprehensive explanation of how work–family conflict influences the connection between psychological capital and athletic achievement, while recognizing the need for future longitudinal and cross-cultural validation studies. This research addresses two main questions:

1. Can work–family conflict, perceived stress, and psychological capital (including self-efficacy, hope, optimism, and resilience) predict professional athletes’ success and failure?
2. What are the most significant predictors of sports success and failure among professional athletes?

Despite the acknowledged limitations, this study makes several valuable contributions to the sports psychology literature. The research demonstrates empirically supported relationships between psychological constructs and performance outcomes, offering practical insights for athlete development programs. The findings provide a foundation for developing more effective psychological interventions that address both individual psychological resources and the broader social context within which athletes operate, ultimately contributing to more sustainable and successful athletic careers.

## 2 Methodology

This study employed a quantitative correlational design utilizing secondary data analysis to examine the predictive relationships between psychological capital, work–family conflict, perceived stress, and athletic performance outcomes. The research utilized discriminant analysis to identify variables that best differentiate between successful and unsuccessful athletes and to develop a predictive model for group membership.

### 2.1 Participants

The study sample comprised 240 professional athletes drawn from four previously published studies: Dewi et al. (2020); Ferdowsi et al. (2021); Obrenovic et al. (2020); and Yan et al. (2022). Participants were selected through purposive sampling to ensure representation of both successful and unsuccessful athletes at the championship level.

#### 2.1.1 Demographic characteristics

Age: Participants ranged from 18 to 35 years (M = 24.5, SD = 4.2 years). The age distribution was comparable between successful (M = 24.7, SD = 4.1) and unsuccessful (M = 24.3, SD = 4.3) athlete groups.

Gender: The sample included 144 male athletes (60%) and 96 female athletes (40%). Gender distribution was balanced between groups, with 72 males and 48 females in each performance category.

Sport Categories: Participants represented various sports disciplines including individual sports (athletics, swimming, gymnastics; *n* = 96, 40%), team sports (football, basketball, volleyball; *n* = 84, 35%), and combat sports (wrestling, boxing, martial arts; *n* = 60, 25%).

Competition Level: All participants competed at national or international levels, with 180 athletes (75%) having national-level experience and 60 athletes (25%) having international-level experience.

#### 2.1.2 Group classification

Athletes were classified into two performance categories based on objective competition results:

Successful Athletes (*n* = 120): Athletes who achieved first, second, or third place finishes in national, regional, or international competitions within the past 2 years. Classification was verified through official sports committee records and performance documentation. Unsuccessful Athletes (*n* = 120): Athletes who participated in championship-level competitions but did not achieve podium finishes (4th place or lower) in national, regional, or international competitions within the same timeframe.

#### 2.1.3 Sampling rationale

Purposive sampling was employed to ensure adequate representation of both performance categories and to maintain comparability across groups. This sampling approach was necessary to: (a) ensure sufficient statistical power for discriminant analysis, (b) maintain homogeneity in competitive level while allowing for performance differentiation, and (c) enable meaningful comparison between success and failure groups while controlling for competition level.

### 2.2 Measures

#### 2.2.1 Work–family conflict scale

Work–family conflict was assessed using the Carlson et al. (2000) Work–Family Conflict Scale. This 12-item instrument measures bidirectional work–family conflict using a 5-point Likert scale (1 = strongly disagree, 5 = strongly agree). The scale comprises two 6-item subscales: (a) Work-to-Family Conflict (sample item: “The demands of my athletic career interfere with my family life”) and (b) Family-to-Work Conflict (sample item: “Family demands interfere with my athletic training and competition”). Higher scores indicate greater work–family conflict. The original scale demonstrated strong reliability (*α* = 0.87; Çarıkçı et al., 2002) and has been validated across diverse populations.

#### 2.2.2 Perceived stress scale

Perceived stress was measured using Cohen et al. (1983) Perceived Stress Scale (PSS). This 14-item instrument assesses the degree to which individuals perceive their lives as unpredictable, uncontrollable, and overwhelming using a 5-point Likert scale (0 = never, 4 = very often).

Sample items include “How often have you felt nervous and stressed?” and “How often have you felt that things were going your way?” (reverse scored). Total scores range from 0 to 56, with higher scores indicating greater perceived stress. The scale demonstrated good reliability (α = 0.76; Cohen et al., 1983) and has been extensively validated across diverse populations.

#### 2.2.3 Psychological capital questionnaire

Psychological capital was assessed using the Luthans et al. (2007) Psychological Capital Questionnaire (PCQ). This 24-item instrument measures four dimensions of psychological capital using a 6-point Likert scale (1 = strongly disagree, 6 = strongly agree): Hope (6 items): Measures goal-directed energy and pathways thinking (sample item: “I can think of many ways to reach my current athletic goals”).

Optimism (6 items): Assesses positive attributional style about current and future success (sample item: “I’m optimistic about what will happen to me in the future as it pertains to my athletic career”).

Resilience (6 items): Evaluates capacity to bounce back from adversity (sample item: “I can get through difficult times in my athletic career because I’ve experienced difficulty before”). The neurobiological mechanisms of such resilience are complex; for example, studies on fear extinction have shown that the activation of specific neural pathways in the prefrontal cortex is critical for regulating emotional responses and recovering from adverse experiences (Zou et al., 2024).

Self-Efficacy (6 items): Measures confidence in one’s ability to execute specific tasks (sample item: “I feel confident in representing my work area in meetings with other athletes”). This confidence is a key component of an individual’s motivational and behavioral toolkit, which can be strategically deployed in social contexts, such as using ingratiation behaviors to navigate challenging interpersonal dynamics like workplace exclusion (Zhang H, et al., 2025). Total psychological capital scores range from 24 to 144, with higher scores indicating greater psychological capital. The scale demonstrated excellent reliability (*α* = 0.89; Luthans et al., 2007) and has been validated across various performance contexts.

#### 2.2.4 Psychometric properties and validation

All instruments underwent additional validation through expert review to ensure appropriateness for the athletic context. A panel of 10 experts in sports psychology and management reviewed the questionnaires for content validity. The expert panel comprised: 4 sports psychologists with doctoral degrees and minimum 10 years of experience working with elite athletes, 3 sports management professionals with advanced degrees and championship-level coaching experience, 3 academic researchers specializing in sports psychology and athlete development The validation process involved: (a) independent review of item relevance and clarity, (b) assessment of cultural appropriateness for the target population, (c) evaluation of construct coverage and representation, and (d) consensus meeting to address any identified concerns. All instruments achieved acceptable content validity with inter-rater agreement exceeding 0.80.

### 2.3 Procedure data

Collection procedures varied across the four source studies but followed similar protocols. Athletes were approached through their respective sports organizations and provided informed consent before participation. Questionnaires were administered in group settings or individually, depending on athletes’ schedules and preferences. Data collection occurred during non-competition periods to minimize performance-related stress influences. All participants completed the questionnaires within a standardized timeframe to ensure consistency. For the current secondary analysis, data were compiled from the four source studies, with careful attention to maintaining participant anonymity and ensuring consistent variable coding across datasets. Data quality checks were performed to identify and address any missing values or outliers.

### 2.4 Data analysis

Statistical analyses were conducted using SPSS version 28.0. The analytical approach included: (a) descriptive statistics: Means, standard deviations, and frequency distributions were calculated for all variables to characterize the sample and assess data distribution, (b) preliminary analyses: Assumptions for discriminant analysis were tested, including multivariate normality, homogeneity of variance–covariance matrices, and absence of multicollinearity. Missing data patterns were examined and addressed through appropriate methods, (c) iscriminant analysis: Stepwise discriminant analysis was employed to identify variables that best differentiate between successful and unsuccessful athletes. This technique determines which combination of predictor variables maximally separates the two groups while developing a discriminant function for group classification, (d) model validation: Cross-validation procedures were implemented to assess the stability and generalizability of the discriminant function. Classification accuracy was evaluated through confusion matrices and hit ratios, and (e) effect size calculations: Practical significance was assessed through calculation of effect sizes and examination of structure matrix coefficients to determine the relative importance of predictor variables. Alpha level was set at 0.05 for all statistical tests, with appropriate adjustments for multiple comparisons where applicable.

## 3 Results

Prior to conducting discriminant analysis, data were examined for violations of statistical assumptions. Tests for multivariate normality, homogeneity of variance–covariance matrices, and multicollinearity were satisfactory. No extreme outliers were identified, and missing data were minimal (< 2%) and addressed through list wise deletion.

### 3.1 Descriptive statistics and group differences

Table 1 presents descriptive statistics for all study variables by performance group. To facilitate comparison across measures with different scale ranges, both raw scores and standardized z-scores are reported.

**Table 1:** Descriptive statistics and group comparisons.

variables	Successful athletes (*n* = 120)	Unsuccessful athletes (*n* = 120)	Group comparison
	M (SD)	Z-Score	M (SD)
Psychological capital			
Hope	28.45 (3.21)	0.42	24.12 (4.18)
Optimism	27.89 (2.87)	0.38	23.76 (3.92)
Resilience	26.73 (3.45)	0.35	22.18 (4.02)
Self-efficacy	29.12 (2.98)	0.41	24.89 (3.76)
Total PsyCap	112.19 (8.92)	0.39	94.95 (12.43)
Work–family conflict			
Work-to-family	18.76 (4.23)	−0.31	22.45 (5.12)
Family-to-Work	17.89 (3.87)	−0.28	21.23 (4.94)
Total WFC	36.65 (7.45)	−0.30	43.68 (9.23)
Perceived stress	21.34 (6.78)	−0.25	26.87 (8.21)

PsyCap, Psychological Capital; WFC, Work–Family Conflict; Cohen’s d effect sizes: small = 0.20, medium = 0.50, large = 0.80. Results indicate significant differences between successful and unsuccessful athletes across all measured variables. Successful athletes demonstrated significantly higher psychological capital across all dimensions (large effect sizes, d > 0.80) and significantly lower work–family conflict and perceived stress (medium to large effect sizes).

### 3.2 Discriminant analysis results

#### 3.2.1 Variable selection and function development

Stepwise discriminant analysis was conducted to identify variables that best differentiate between successful and unsuccessful athletes. The analysis employed Wilks’ lambda criterion for variable entry (*p* < 0.05) and removal (*p* > 0.10; Table 2).

**Table 2:** Stepwise discriminant analysis summary.

Step	Variable entered	Wilks’ lambda	F to enter	df	p	Cumulative R^2^
1	Self-Efficacy	0.847	42.78	1,238	< 0.001	0.153
2	Optimism	0.789	31.25	2,237	< 0.001	0.211
3	Hope	0.743	27.92	3,236	< 0.001	0.257
4	Work–Family Conflict	0.718	23.16	4,235	< 0.001	0.282
5	Resilience	0.701	19.84	5,234	< 0.001	0.299

Variables are listed in order of entry. Perceived stress did not meet entry criteria (F = 2.14, *p* = 0.145) after controlling for other variables in the model.

Perceived stress was excluded from the final discriminant function because its predictive contribution became non-significant once the psychological capital dimensions and work–family conflict were included in the model, suggesting that its effects are mediated through these other variables.

#### 3.2.2 Discriminant function characteristics

The discriminant analysis produced one significant function (*Λ* = 0.701, χ^2^ = 83.42, df = 5, *p* < 0.001) that explained 29.9% of the variance between groups. This function was labeled the “Positive Resource-Stress Balance Function” based on the pattern of variable contributions (Table 3).

**Table 3:** Discriminant function coefficients and structure matrix.

Variable	Standardized coefficients	Structure matrix	Group centroids
Self-efficacy	0.421	0.847	
Optimism	0.389	0.808	
Hope	0.356	0.783	
Work–family conflict	−0.298	−0.563	
Resilience	0.287	0.768	
Group centroids			
Successful athletes			1.12
Unsuccessful athletes			−1.12

Structure matrix coefficients represent correlations between variables and the discriminant function. Coefficients ≥ |0.30| are considered meaningful contributors.

#### 3.2.3 Classification accuracy

The discriminant function demonstrated strong classification accuracy. Cross-validation procedures revealed that 78.3% of cases were correctly classified, with 81.7% of successful athletes and 75.0% of unsuccessful athletes correctly identified. This classification accuracy significantly exceeded chance level (50%) and indicates good practical utility of the model (Table 4).

**Table 4:** Classification results.

Predicted group membership		
Actual group	Successful	Unsuccessful
Successful	98 (81.7%)	22 (18.3%)
Unsuccessful	30 (25.00%)	90 (75.00%)

Cross-validated classification results. Percentages represent hit rates for each group.

The results provide support for the hypothesized relationships between psychological capital, work–family conflict, and athletic performance outcomes. The discriminant analysis identified a meaningful pattern where successful athletes are characterized by higher levels of psychological capital (particularly self-efficacy, optimism, and hope) and lower work–family conflict compared to their unsuccessful counterparts. The predominance of psychological capital dimensions in the discriminant function (four of five variables) underscores the importance of positive psychological resources in athletic success. The inclusion of work–family conflict as a significant predictor highlights the often-overlooked role of life balance in athletic performance, supporting the study’s theoretical framework regarding the interaction between personal resources and environmental stressors. The exclusion of perceived stress from the final model suggests that while stress differences exist between groups, these differences may be better explained through the lens of available psychological resources and life balance factors rather than as an independent predictor.

## 4 Discussion

This study aimed to explore how work–family conflict, perceived stress, and dimensions of psychological capital namely self-efficacy, hope, optimism, and resilience predict sports success and failure among professional athletes. The findings showed that five out of six predictor variables optimism, self-efficacy, hope, work–family conflict, and resilience significantly distinguished between successful and unsuccessful athletes. These results support the proposed hypothesis that psychological resources and life stressors interact to influence athletic performance. Specifically, higher levels of psychological capital were associated with greater sporting success, aligning with the principles of the positive psychology framework in performance settings.

Optimism as a predictor of athletic success: Luthans and Youssef-Morgan (2017) highlight that optimistic athletes are more likely to perceive fairness and respect within their sport environments, which reinforces their positive outlook. This aligns with the present findings that optimism significantly predicts athletic success. According to Bhat (2017), optimistic athletes persist longer and remain more motivated during training setbacks. In contrast, athletes lacking optimism may become disengaged, distrustful toward team leadership, and susceptible to performance decline (Dirmanchi and Khanjani, 2019). This cognitive bias toward expecting positive outcomes appears to be a key factor distinguishing high performers from their less successful peers.

Self-Efficacy and performance excellence: Sivrikaya (2018) emphasizes that self-efficacy enhances athletes’ sense of control, enabling them to persist through difficult conditions and influence match outcomes. Athletes with stronger self-efficacy set more ambitious goals and engage in adaptive coping strategies under pressure. These findings suggest that successful athletes differ from unsuccessful ones in their ability to translate confidence into consistent high-level performance. The “fighting spirit” aspect of self-efficacy motivates athletes to frame challenges as growth opportunities rather than threats.

Hope as a motivational resource: Bhat (2017) notes that hopeful athletes display greater goal focus and motivation. The current findings reinforce that hope especially the ability to generate alternative strategies toward goals (pathway thinking) plays a crucial role in distinguishing successful athletes. Minda and Piasecka (2019) found that hopeful athletes manage stress more effectively and perceive their support systems as reliable. In contrast, athletes with low hope are more vulnerable to fatigue, disengagement, and performance dips. Hope appears to function as a forward-looking motivational engine, particularly relevant under competitive pressure.

Work–Family conflict and performance outcomes: Taylor et al. (2019) describe how work–family conflict imposes emotional strain on athletes by forcing them to juggle professional obligations with personal commitments. The present study found that unsuccessful athletes reported higher levels of work–family conflict, indicating that role interference can undermine performance. Zheng and Wu (2018) suggest that successful athletes more effectively compartmentalize these roles, preserving emotional stability and minimizing stress. The ability to manage competing demands on and off the field may represent a key differentiator of athletic success.

Resilience and Adaptive Capacity: Resilient athletes bounce back faster from adversity, maintaining emotional balance and performance stability. Gould et al. (2001) identify resilience as essential for coping with pressure and sustaining self-esteem. The present findings suggest that successful athletes possess stronger resilience profiles, enabling them to recover from setbacks and avoid performance declines. Unsuccessful athletes, in contrast, may struggle to re-establish momentum following failure or disruption.

Perceived stress: a complex relationship: Despite its theoretical relevance, perceived stress did not significantly differentiate successful from unsuccessful athletes in this study. Sanıoglu et al. (2017) clarify that perceived stress as measured by tools like Cohen’s Perceived Stress Scale captures chronic psychological strain rather than acute arousal. This distinction may explain the null findings. According to Örencik et al. (2023), successful athletes develop stable psychosocial resource profiles that buffer the impact of perceived stress through compensatory mechanisms. Their longitudinal research indicates that resource interdependencies not stress levels alone are key predictors of performance stability. Thus, it may not be stress per se, but how athletes regulate and respond to it, that determines success (Kim et al., 2017).

This study’s cross-sectional design limits causal inference, and varying definitions of athletic “success” may affect generalizability across sports and cultures. Findings may also differ in non-elite or amateur populations. Despite these constraints, the results support incorporating psychological capital development particularly optimism, hope, self-efficacy, and resilience into athlete training. Addressing work–family conflict through structured interventions can further enhance performance consistency. Future studies should adopt longitudinal designs and explore how athletes build and apply psychological resources over time, including cultural and contextual influences on stress adaptation and resource development.

## 5 Future work

In this article, a quantitative correlational design utilizing secondary data analysis was used and produced acceptable results. However, for studies such as Satellite and Spacecraft Operations (Xiao et al., 2024; Ye et al., 2024), Airport and Air Traffic Management (Wang et al., 2024), Welding Microstructure and Mechanical Behavior (Bao et al., 2024; Du et al., 2024; Lv et al., 2024), Pain and Inflammation Studies (Dai et al., 2020; Li X.-Y, et al., 2023; Li X, et al., 2023; Liao et al., 2024; Lin et al., 2020; Lin et al., 2024), Clinical and Diagnostic Studies (Wu et al., 2024; Wu et al., 2009; Yang, 2023; Zhengzhi et al., 2011), Traditional and Plant-based Medicine (Li-chun et al., 2020; Xiang X, et al., 2025; Zhao et al., 2011), AUV and Image Processing (Xiang D, et al., 2025; Zhang et al., 2024), Debris Flow and Natural Hazards (Zhiquan et al., 2015; Zhiquan et al., 2016), Transmission and Friction (Gao, 2017), Transportation Safety (Yi et al., 2024), Deep Learning and Image Analysis (Guan et al., 2025; Yu et al., 2025), Networking and Edge Computing (Zheng and Cao, 2024), Minimally Invasive Surgery (Zhang M.-X, et al., 2025; Zhang M, et al., 2025), Human Behavior & Genetics (Hou et al., 2022), Climate Policy and Carbon Neutrality (Li B, et al., 2025; Wang et al., 2025a; Wang et al., 2025b; Yu et al., 2023; Yu et al., 2024), Urban Green Space and Ecology (Qindong et al., 2025), Plant Immunity and Resistance (Ding et al., 2024; Wu et al., 2025), Rail Systems (Chen et al., 2025), Travel and Economic Development (Huang et al., 2025), it is recommended to use this method if suitable data are available.

## 6 Conclusion

This study underscores the pivotal role of psychological capital and work-family dynamics in shaping athletic success. Among the dimensions examined, optimism and self-efficacy emerged as the most robust predictors of superior athletic performance, reflecting the critical importance of internal psychological resources in sustaining motivation, resilience, and adaptive functioning under pressure. Hope and resilience further contributed to distinguishing successful athletes, reinforcing the value of psychological capital as a foundational element in elite performance environments. In contrast, work–family conflict was found to significantly hinder performance outcomes, suggesting that athletes who experience greater role strain between professional and personal domains may have diminished psychological capacity to cope with competitive demands. This finding highlights the need for integrated support systems that help athletes manage dual-role pressures effectively. Interestingly, perceived stress did not emerge as a significant direct predictor of success. This may reflect the complex and potentially curvilinear relationship between stress and performance, in which both under- and over-arousal can impair functioning. Instead, athletes’ psychological capital may act as a compensatory mechanism that buffers the effects of stress on performance, aligning with recent person-oriented perspectives on stress regulation in elite sport. These findings have several applied implications. Sport psychologists, coaches, and performance staff are encouraged to incorporate psychological capital development especially in optimism and self-efficacy into regular training programs. Furthermore, addressing work-family balance through psychoeducation and organizational flexibility may enhance athletes’ overall well-being and competitive consistency. The study adds conceptual value by positioning work–family conflict not merely as an external stressor, but as a moderating force that shapes the functional impact of internal psychological strengths. Overall, this research contributes to a growing body of evidence emphasizing the interplay between personal and contextual resources in sport psychology. By highlighting psychological capital and work–family conflict as key differentiators between successful and less successful athletes, the study offers both theoretical insight and actionable direction for future interventions targeting sustainable high performance.

## Data Availability

The original contributions presented in the study are included in the article/supplementary material, further inquiries can be directed to the corresponding author.
